# Development and validation of the CAIL prognostic score in non‐small cell lung cancer patients with malignant pleural effusion

**DOI:** 10.1111/crj.13700

**Published:** 2023-09-18

**Authors:** Tianyuan Li, Panwen Tian, Qin Huang, Hao Zeng, Qi Wei, Yalun Li

**Affiliations:** ^1^ Department of Pulmonary and Critical Care Medicine, State Key Laboratory of Respiratory Health and Multimorbidity, Precision Medicine Key Laboratory of Sichuan Province West China Hospital, Sichuan University Chengdu Sichuan China; ^2^ Lung Cancer Center, West China Hospital Sichuan University Chengdu Sichuan China

**Keywords:** malignant pleural effusion, non‐small cell lung cancer, overall survival, prognostic score

## Abstract

**Background:**

Patients with malignant pleural effusion (MPE) typically have poor prognoses, and predicting survival is challenging. The present study aimed to identify prognostic factors of overall survival (OS) in non‐small cell lung cancer (NSCLC) patients with MPE in the time of immunotherapy and targeted therapy.

**Methods:**

Data of 344 consecutive NSCLC patients with MPE on clinical, radiological, and molecular characteristics and treatment options were collected. The risk factors in the training cohort were assessed using univariate and multivariate proportional hazards analyses. A clinical prognostic score was established and validated.

**Results:**

According to the results of the multivariable survival analysis, the Eastern **C**ooperative Oncology Group (ECOG) performance score (PS), **a**ntiangiogenic therapy, **i**mmunotherapy, and **l**actic dehydrogenase (LDH) in pleural fluid (CAIL) prognostic score was developed (*n* = 275) and subsequently validated (*n* = 69). Patients who underwent risk stratification into low‐, moderate‐, and high‐risk groups had median OS of 46.1, 23.1, and 9.6 months, respectively (*P* < 0.0001). The area under the curve (AUC) analysis showed the CAIL score to be superior at predicting survival compared with the LENT score at 6 (0.84 vs. 0.77, *P* < 0.01), 12 (0.87 vs. 0.82, *P* < 0.01), and 36 months (0.80 vs. 0.77, *P* < 0.01).

**Conclusions:**

For NSCLC patients with MPE, the validated CAIL prognostic score integrates clinical characteristics and therapeutic modalities to predict survival.

## Graphical abstract

The Eastern **C**ooperative Oncology Group (ECOG) performance score (PS), **a**ntiangiogenic therapy, **i**mmunotherapy, and **l**actic dehydrogenase (LDH) in pleural fluid (CAIL) prognostic score was to predicted the survival of NSCLC patients with MPE. The prognostic score was shown to be easy to calculate and practical to use in clinical work and valuable for clinicians in selecting optimal management strategies.

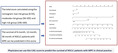

## 1 INTRODUCTION

Malignant pleural effusion (MPE) is typically regarded as a poor prognostic factor and is associated with a median survival time of between 3 and 12 months, based on the comorbidities of the patient and the underlying malignancy.^1^, ^2^ Palliation of symptoms, avoiding needless pleural procedures, reducing the need for hospitalization, and shortening hospital stays when necessary are the management goals for patients with MPE.^3^ In the past 10 years, the variety of treatment options and pleural procedures for MPE has expanded, resulting in increased patient categorization and therapy individualization but also to a variety of outcomes, such the success and survival of pleurodesis success and survival.^4^

Previously, the LENT scoring system was developed in 2014, which predicted patient survival according to pleural fluid lactate dehydrogenase (LDH), Eastern Collaborative Oncology Group (ECOG) performance status (PS) score, type of tumor, and blood neutrophil‐to‐lymphocyte ratio (NLR), and it was the first validated prognostic model in MPE but has not been prospectively assessed.^2^ In addition, the first validated risk stratification scoring system, the PROMISE model, including a discovered pleural fluid biomarker (TIMP1) with clinical data (previous radiotherapy or chemotherapy, hemoglobin, leukocyte, C‐reactive protein [CRP], PS, and type of primary cancer), was utilized for the prospective evaluation of the survival of patients with MPE.^5^ However, the PROMISE score took into account biological variables, which would restrict its usefulness in all medical settings.

Recently, immunotherapy and targeted therapy have dramatically improved the prognosis of advanced lung cancer. Immunotherapy has shown benefits as an exciting treatment for advanced non‐small cell lung cancer (NSCLC) patients without actionable oncogenic drivers. It significantly prolongs patient survival compared with chemotherapy or radiotherapy.^6^ Additionally, targeted therapies have also achieved sustained improvements in survival in patients with NSCLC who have actionable mutations.^7^ However, neither the LENT score or PROMISE score included patients' therapy patterns, and these scores forecasted the prognosis of every malignant tumor, which made less accurate of predicting the prognosis of a particular tumor type.

The current study's objective was to develop a survival score combining the therapy modality and clinicopathological features to predict the prognosis of NSCLC patients with MPE and to show how the prognostic factors and overall survival (OS) are related.

## 2 MATERIALS AND METHODS

### 2.1 Participants

A retrospective cohort analysis of consecutive NSCLC patients who previously had a thoracentesis for MPE was conducted at West China Hospital of Sichuan University between June 2010 and June 2021. The following were the inclusion criteria: (1) at least 18 years old; (2) pathologically confirmed NSCLC; (3) histological or cytological confirmation of MPE; (4) received systemic treatment following the initial MPE event; and (5) all clinical information and follow‐up data accessible. Patients with other tumors and with incomplete data were excluded. The West China Hospital's Ethics Committee gave its approval to this study (No. 2022‐1085), and the project was carried out in compliance with the 2013 revision of the Declaration of Helsinki.

### 2.2 Data collection

Data on each patient's clinical traits, laboratory results, and treatment details were taken from the electronic inpatient record system. Clinical characteristics included sex, age, smoking status, pathology, ECOG PS, metastatic sites, and driver mutation. Baseline peripheral blood indicators included CRP, white blood cells (WBCs), serum albumin, hemoglobin, LDH, and NLR. Additionally, location of pleural fluid and LDH level in pleural fluid were included. Treatment records included local pleural therapy and systemic treatment strategies, which included targeted therapy, immunotherapy, chemotherapy, and antiangiogenic therapy. The time from diagnosis to death from any cause or the last date the patient was known to be alive was defined as OS.

### 2.3 Development and validation of the score

The patients were divided into a training cohort and an internal validation group in a 7:3 ratio using computer‐generated random numbers. We used K‐fold cross validation to divide the dataset into 5 equally sized sample subsets, using a concatenation of 4 subsets as the training cohort and the remaining one as the validation cohort each time, performing 5 training and validation runs. The final evaluation metric is the average of the five evaluations.

$${\mathrm{CV}}_{\left(k\right)}=\frac{1}{k}\sum_{i=1}^{k}{\mathrm{MSE}}_{i}.$$

The impact of clinical characteristics, laboratory parameters, and treatment modality on OS in the training cohort were assessed using univariate and multivariate proportional hazards models. Depending on the total number of risk factors, the X‐tile software determined the cutoff point, which was used to divided patients from the training cohorts and validation cohorts into low‐, moderate‐, and high‐risk groups. The receiver operating characteristic (ROC) curve was used to test the sensitivity and specificity of the prognostic score in the training and validation cohorts for 6‐, 12‐, and 36‐month OS as binary outcomes. In addition to obtaining the area under the curve (AUC) evaluation metric, we also produced prediction model calibration curves. Predicted probability was plotted on the *x*‐axis. The gray dashed line showed the ideal calibration line. The red solid line means the performance of the model, and the closer it is to the ideal line, the better the prediction. As shown in Figure S1, the predicted and actual values for the test and validation cohorts fluctuated less and performed well. Finally, we compared the current score with the LENT score.

### 2.4 Statistical analysis

The prognostic values of the predictors were determined using Cox regression, and the findings are shown as hazard ratios (HRs) and 95% confidence intervals (CIs). The nomogram was created using predictions obtained from the multivariate Cox regression analysis, and it was then confirmed. To evaluate survival, Kaplan–Meier curves and the log‐rank test were employed. The AUC and concordance index (C‐index) were employed to assess the capacity of nomogram for discrimination. In the univariate analysis, variables having a *P* value less than 0.1 were included in the multivariate analysis. Statistical significance was defined as a two‐tailed *P* value 0.05. R studio (version 2021.09.0 for Mac) was used to conduct all statistical analyses.

## 3 RESULTS

### 3.1 Demographic characteristics of NSCLC patients with MPE

In total, 344 NSCLC patients with MPE were included. The mean age was 61.4 years, 54.3% (187) were males, and 63.6% (219) were never smokers. A total of 81.6% (281) of patients were identified as lung adenocarcinoma, and 18.3% (63) were diagnosed with other histological types. A total of 57.8% (199) of patients harbored actionable mutations, involving *EGFR* mutations, *ALK* fusion, or *ROS1* fusion. Totally, 58.4% of patients received molecular targeted therapy, and 24.2%, 12.2%, and 46.5% received immunotherapy, antiangiogenic therapy, and chemotherapy, respectively.

Of the 344 patients, 275 individuals were assigned to the training cohort, and the leaving 69 patients were assigned to the validation cohort. The demographic characteristics and laboratory parameters are shown in (Table 1).

**TABLE 1:** Demographic characteristics of non‐small cell lung cancer patients with MPE.

Characteristics	Total (*n* = 344)	Training set (*n* = 275)	Validation set (*n* = 69)
Age, median, (25th, 75th)	61.4 (52.9,69.3)	62.0 (53.2,69.5)	60.3 (52.7,66.4)
Gender, *n* (%)			
Male	187 (54.36)	152 (55.27)	35 (50.72)
Female	157 (45.64)	123 (44.73)	34 (49.28)
Smoking status, *n* (%)			
Never smoker	219 (63.66)	174 (63.27)	45 (65.22)
Smoker	125 (36.33)	101 (36.73)	24 (34.78)
ECOG PS			
0–1	222 (64.53)	171 (62.18)	51 (73.91)
≥2	122 (35.47)	104 (37.82)	18 (25.09)
Histology, *n* (%)			
Adenocarcinoma	281 (81.69)	226 (82.18)	55 (79.71)
Other NSCLC	63 (18.31)	49 (17.82)	14 (20.29)
History of cardiovascular disease			
No	229 (66.57)	178 (64.73)	51 (73.91)
Yes	115 (33.43)	97 (35.27)	18 (26.09)
Laterality of pleural effusion			
Left	136 (39.53)	110 (40.00)	26 (37.68)
Right	208 (60.47)	165 (60.00)	43 (62.32)
Metastatic, *n* (%)			
Brain	41 (11.91)	29 (10.55)	12 (17.39)
Liver	21 (6.10)	17 (6.18)	4 (5.80)
Bone	87 (25.30)	69 (25.09)	18 (26.09)
Adrenal	18 (5.23)	15 (5.45)	3 (4.35)
Contralateral lung	90 (26.16)	72 (26.18)	18 (26.09)
Driver mutation			
No	145 (42.15)	117 (42.55)	28 (40.58)
Yes	199 (57.85)	158 (57.45)	41 (59.42)
Fluid LDH, IU/L			
<1500	296 (86.05)	233 (84.73)	63 (91.30)
≥1500	48 (13.95)	42 (15.27)	6 (8.70)
NLR			
<9	316 (91.86)	255 (92.72)	61 (88.41)
≥9	28 (8.14)	20 (7.27)	8 (11.59)

*Note*: Data are presented as No. (%) or median (interquartile range) or mean ± SD.

Abbreviations: ECOG PS, Eastern Cooperative Oncology Group performance score; LDH, lactate dehydrogenase; MPE, malignant pleural effusion; NLR, neutrophil‐to‐lymphocyte ratio; NSCLC, non‐small cell lung cancer.

### 3.2 Assessment of prognostic factors in the training cohort

In univariate analysis in the training cohort, smoking status, histology, chemotherapy, targeted therapy, immunotherapy, antiangiogenic therapy, positive driver mutations (*EGFR*, *ALK*, and *ROS1*), ECOG PS, and LDH level in pleural fluid were confirmed to be significant (Table 2).

**TABLE 2:** Univariate and multivariate proportional hazards analyses of OS in the training cohort.

Covariate	Univariate analysis			Multivariable analysis	
	Median survival month	HR (95% CI)	*P*	HR (95% CI)	*P*
Sex					
Male	22.93	1			
Female	22.57	0.867 (0.641,1.171)	0.351		
Age years					
<65	22.57	1			
≥65	20.03	1.057 (0.777,1.437)	0.724		
Smoking status					
Never smoker	23.73	1			
Smoker	18.63	1.366 (1.007,1.852)	0.045		
ECOG PS					
0–1	33.73	1			
≥2	9.63	2.204 (1.914,2.539)	<0.001	6.39 (4.48,9.11)	<0.001
Histology, *n* (%)					
Adenocarcinoma	24.20	1			
Other NSCLC	14.13	1.607 (1.118,2.310)	0.014		
History of cardiovascular disease					
No	24.20	1			
Yes	18.63	1.283 (0.933,1.763)	0.125		
Laterality of pleural effusion					
Left	22.93	1			
Right	22.30	1.066 (0.782,1.453)	0.687		
Metastatic, *n* (%)					
Brain	22.57	1.142 (0.691,1.889)	0.604		
Liver	14.97	1.585 (0.859,2.926)	0.141		
Bone	18.83	1.165 (0.819,1.657)	0.397		
Adrenal	11.16	1.805 (0.977,3.334)	0.059		
Contralateral lung	26.1	0.885 (0.631,1.241)	0.478		
Driver mutation					
No	28.23	1			
Yes	13.77	0.445 (0.328,0.605)	<0.001		
Treatment					
Targeted therapy	27.20	0.488 (0.361,0.660)	<0.001		
Immunotherapy	27.00	0.761 (0.500,1.158)	0.023	0.57 (0.36,0.91)	0.018
Antiangiogenic therapy	27.17	0.661 (0.409,1.068)	0.090	0.50 (0.29,0.83)	0.048
Chemotherapy	18.63	1.374 (1.016,1.859)	0.039		
Fluid LDH, IU/L					
<1500	28.83	1			
≥1500	11.40	2.992 (1.995,4.489)	<0.001	2.89 (1.90,4.41)	<0.001
NLR					
<9	23.13	1			
≥9	11.60	1.825 (1.104,3.016)	0.030		

Abbreviations: ECOG PS, Eastern Cooperative Oncology Group performance score; LDH, lactate dehydrogenase; NLR, neutrophil‐to‐lymphocyte ratio; NSCLC, non‐small cell lung cancer; OS, overall survival.

In multivariate analysis, receiving antiangiogenic therapy or immunotherapy was associated with a decreased hazard of survival, and ECOG PS ≥ 2 and LDH level in pleural fluid ≥1500 IU/L were associated with increased hazards of survival. However, in multivariate analysis, positive driver mutation and receiving targeted therapy were not related to a significant HR of survival (Table 2).

### 3.3 Development and calculation of the CAIL score

**FIGURE 1:**
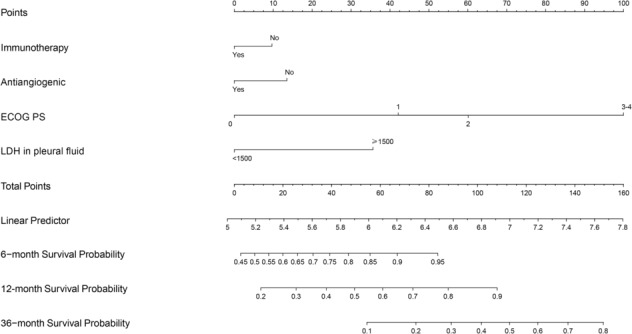
Prognostic nomogram for non‐small cell lung cancer patients with malignant pleural effusion (MPE). The points for each variable were added up to obtain the total points, and the final scores were used to estimate 6‐, 12‐, and 36‐month survival.

Based on the outcomes of the multivariate analysis, four variables (E**C**OG PS, **a**ntiangiogenic therapy, **i**mmunotherapy, and **L**DH level in pleural fluid) were selected to establish the prognostic score called the “CAIL score.” According to the predictive factors identified, we established a nomogram to predict the likelihood of survival at 6, 12, and 36 months in patients with lung cancer who have MPE (Figure 1). On the points scale, a proportional number of risk points were given to each prognostic parameter. Delineating a vertical line, then adding the associated risk points for each parameter, will yield a total score, which has a range of 0–160 (Table S1). Finally, a vertical line can be drawn toward the survival probability axis, which might be used to predict the specific probability of survival at 6, 12, and 36 months for NSCLC patients with MPE.

**FIGURE 2:**
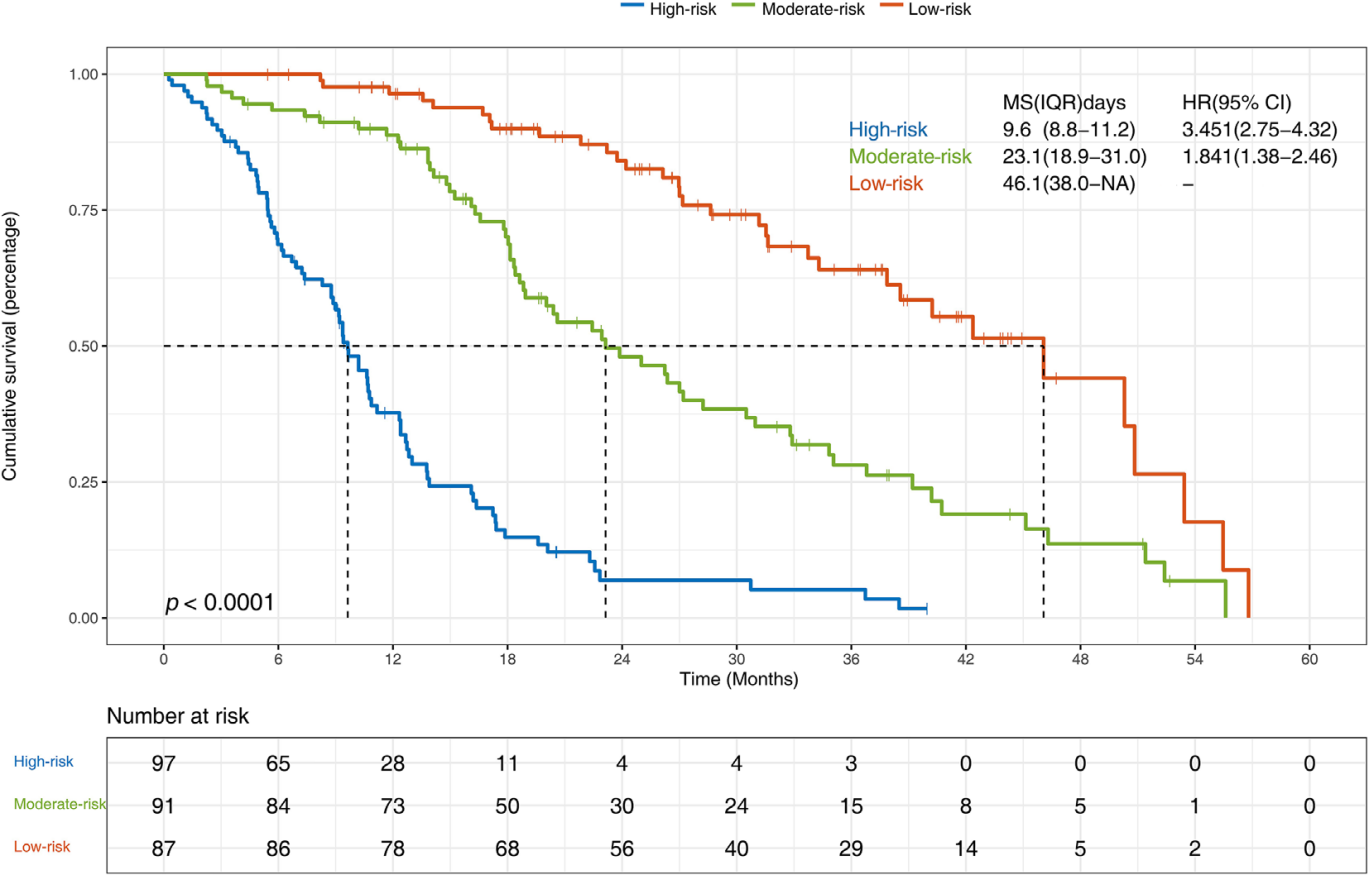
Kaplan–Meier curves of overall survival (OS) in non‐small cell lung cancer patients with malignant pleural effusion (MPE) in training cohort (*P* < 0.0001, log‐rank test).

**FIGURE 3:**
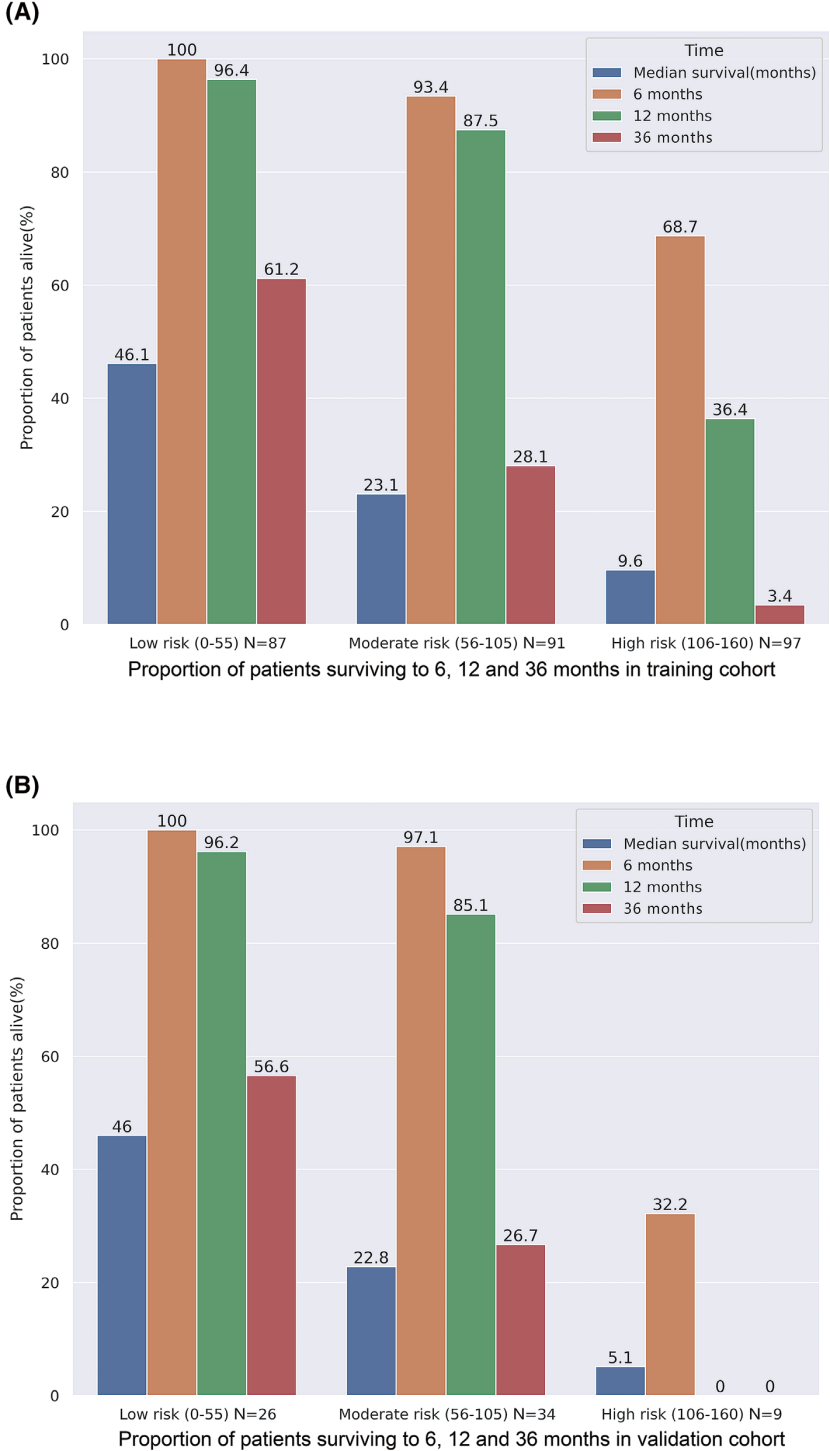
Proportion of patients surviving to 6, 12, and 36 months according to low‐risk, moderate‐risk, and high‐risk CAIL scores. (A) Training cohort; (B) validation cohort.

In addition, patients from the training cohort and validation cohort were split into three subgroups depending on the threshold value of the total score calculated using the nomogram: low‐risk group (0–55), moderate‐risk group (56–105), and high‐risk group (106–160). Of the 275 patients in the training cohort, 87 patients were divided into the low‐risk group, with 100.0%, 96.4%, and 61.2% surviving at 6, 12, and 36 months, respectively, and a median OS of 46.1 months. The median OS of the 91 patients in the moderate‐risk group was 23.1 months, with 93.4%, 87.5%, and 28.1% surviving to 6, 12, and 36 months, respectively. However, the median OS of the 97 patients in the high‐risk group was only 9.6 months (8.8–11.2), with 68.7%, 36.4%, and 3.4% surviving to 6, 12, and 36 months, respectively (Figures 2 and 3A).

In the validation cohort, 26 patients were divided into the low‐risk group, whereas 34 patients and 9 patients were assigned to the moderate‐risk group and the high‐risk group, respectively. Of the 26 patients in the low‐risk group, 100.0%, 96.2%, and 56.6% survived to 6, 12, and 36 months, respectively, with a median OS of 46.0 months (32.2‐NA). The median OS for the 34 patients at the moderate‐risk group was 22.8 months (15.7–33.9), with 97.1% surviving to 6 months, 85.1% surviving to 12 months, and 26.7% surviving to 36 months. Additionally, the median OS for the 9 patients at the high‐risk group was only 5.1 months (4.7‐NA), with 33.2%, 0.0%, and 0.0% surviving to 6, 12, and 36 months, respectively (Figures 2 and 3B).

### 3.4 Validation of the CAIL score

**FIGURE 4:**
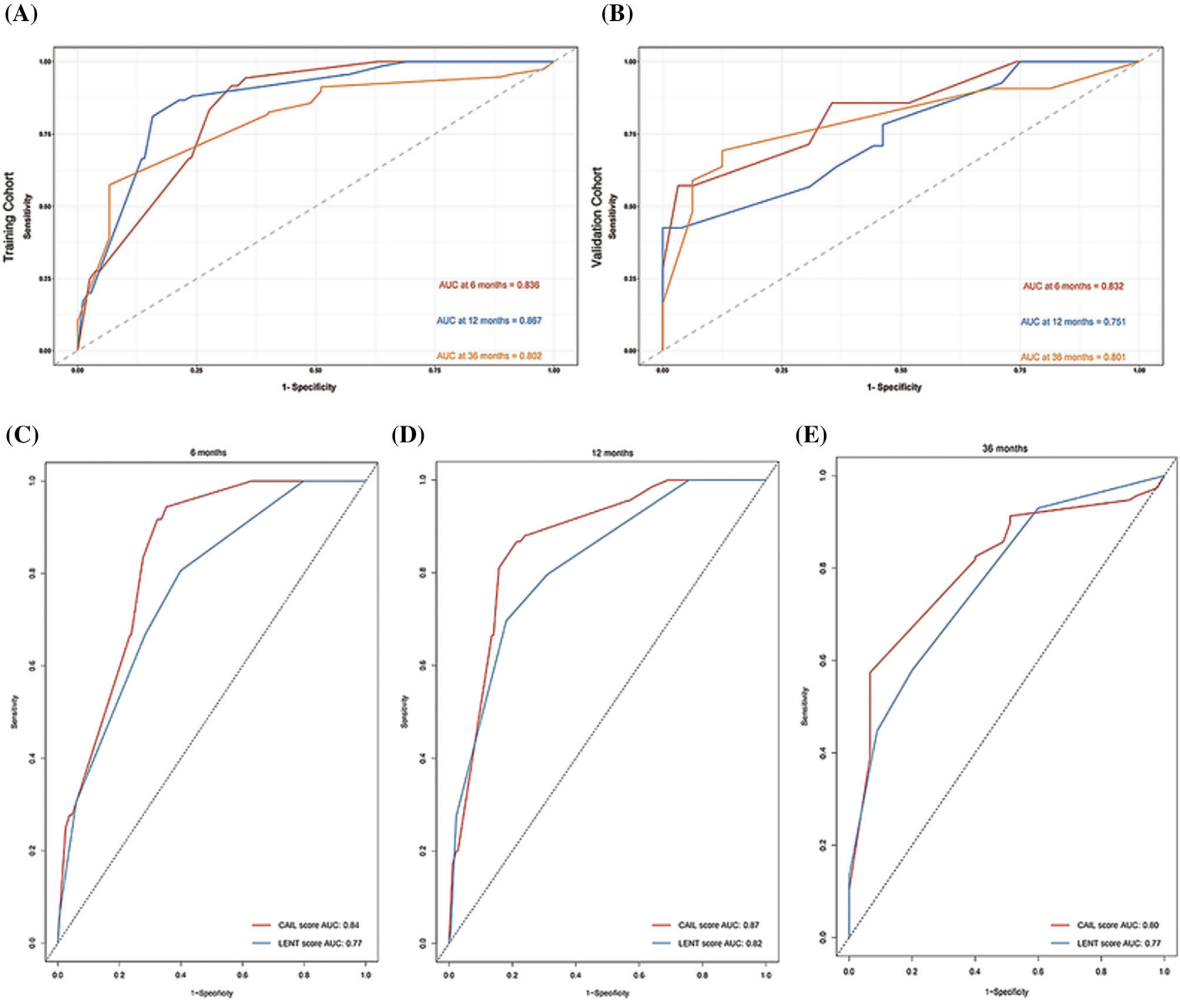
Receiver operating characteristic (ROC) curves for the Eastern **C**ooperative Oncology Group (ECOG) performance score (PS), **a**ntiangiogenic therapy, **i**mmunotherapy, and **l**actic dehydrogenase (LDH) in pleural fluid (CAIL) score at 6, 12, and 36 months: (A) training cohort; (B) validation cohort; and ROC curves of the CAIL score and the LENT score in the training cohort: (C) 6‐, (D) 12‐, and (E) 36 months of overall survival (OS).

The predictive ability of the CAIL score was assessed using ROC analysis. The C‐index for the training cohort was 0.82. The AUC values for the CAIL score were 0.836, 0.867, and 0.802 at 6, 12, and 36 months, respectively (Figure 4A). The C‐index for the validation cohort was 0.78. The AUC values for the validation cohort were 0.832, 0.751, and 0.801 at 6, 12, and 36 months, respectively (Figure 4B). The results of the validation cohort show the high specificity and sensitivity of the CAIL score.

### 3.5 Comparison of the CAIL score with the LENT score in the training cohort

ROC curves were created to compare the 6‐, 12‐ and 36‐month survival predictions of the LENT and the CAIL scores for patients with MPE in the training cohort. At 6, 12, and 36 months, the AUCs were 0.84, 0.87, and 0.80 for the CAIL score, which were significantly better than those for the LENT score of 0.77, 0.82, and 0.77, respectively (Figure 4C–E).

## 4 DISCUSSION

Our study showed that ECOG PS <2, LDH in pleural fluid ≤1500 IU/L, immunotherapy, and antiangiogenic therapy were associated with better OS, and a score with good discriminatory power to predict OS in NSCLC patients with MPE receiving anti‐tumor treatments was developed.

Nomograms were a reliable methods for quantifying risk and have been used widely in predicting prognosis in cancer, which offered a user‐friendly graphic presentation of the estimated probabilities of an event, such as survival, that was tailored to individual patients.^8^, ^9^ However, there has been no effective nomogram for predicting survival in lung cancer patients with MPE recently. According to the above predictive factors, a nomogram and the prognostic score called the “CAIL score” was established to predict the likelihood of survival at 6, 12, and 36 months in NSCLC patients with MPE. In clinical practice, CAIL score could help clinicians to identify those with high risk and make decision.

Previous studies have showed that PS was related to the prognosis of MPE generated from several cancer types.^10^, ^11^, ^12^ Poor PS indicated limited therapeutic effects and shorter OS.

Similar to the LENT model, our score also showed that LDH level of the pleural fluid was related to survival of lung cancer patient. A previously retrospective study of lung adenocarcinoma with MPE demonstrated that patients with higher pleural LDH (>1500 IU/L) had poorer survival compared with those with lower pleural LDH (≤1500 IU/L),^13^ which was consistent with Zhang's results.^14^ Elevated pleural LDH may be associated with glycolysis over oxidative phosphorylation for energy by cancer cells.^15^ NLR, as an inflammatory marker, has also been proved associated with lung cancer patients with MPE.^16^ However, there was no statistically significant distinction between the groups with high and low NLR in our research using multivariable Cox analysis. Like the LENT score, we found that LDH in pleural fluid ≤1500 was associated with longer OS.

The level of pleural vascular endothelial growth factor (VEGF) is a critical pathological factor in the development and progression of NSCLC patients with MPE.^17^ Studies have shown that bevacizumab is a VEGF inhibitor that was regarded as a promising strategy for improving the management of MPE.^18^ Kitamura et al found that 92.3% MPE patients caused by NSCLC who received bevacizumab combined with chemotherapy as first‐ or second‐line treatment achieved MPE control lasting more than 8 weeks.^19^ Similar results were found in other studies,^20^, ^21^ which analyzed the records of NSCLC patients with MPE who consequently received bevacizumab plus chemotherapy. Correspondingly, two phase II trials evaluating the effectiveness of bevacizumab in the treatment of MPE were performed on Japanese patients^22^, ^23^ and revealed that additional bevacizumab into the treatment protocol enhanced the MPE control rate. In our study, we found that antiangiogenic therapy was associated with a significantly longer OS in lung cancer patients with MPE.

Previous research revealed that MPE in patients is a poor predictor of anti‐PD‐1 antibody effectiveness.^24^, ^25^ However, present clinical trials have shown that MPE may be sensitive to immunotherapy.^26^ Li et al. demonstrated that patients with lung cancer achieved a response rate of 81.37% of MPE after receiving a single dose of immune drugs following chest drainage.^27^ According to a study by Grosu et al.,^28^ there was a strong correlation between the expression of PD‐L1 in histology samples and matching pleural fluid from NSCLC patients, suggesting that if the primary tumor was responsive to anti‐PD‐1 therapy, MPE might also potentially be impacted by this therapy.

There have been several reported prognostic scores for survival in MPE. Molina et al.^29^ developed a BLESS model for breast cancer and lung cancer patients with MPE to estimates of survival probability, but our CAIL score was specifically applicable to NSCLC patients with MPE. Tian et al.^30^ constructed a nomogram for predicting the survival of patients with NSCLC with MPE or malignant pleural pericardial effusion at the first diagnosis based on the SEER database; however, the SEER database did not contain some of the clinicopathological factors and molecular markers that may affect the survival of patients with lung cancer, such as physical status and the EGFR mutation. This might restrict how they are used in prognosis prediction scores. Shi et al.^31^ reported the AL score, which incorporated two enzymes (ALP and LDH) from serum and pleural effusion, and investigated the prognostic value for MPE patients. In addition, Zhang et al.^14^ developed the prognostic scores of RECLS and RECLSAM for individuals with lung cancer who have MPE and lung adenocarcinoma who have MPE. However, the treatment options were not included in these scores, and they lack the validation of the scores. For our CAIL score, we developed it including therapy modality and assessed the sensitivity and specificity in the validation cohort.

The merit of the present study includes the large sample size and the clinical applicability of noninvasive risk factor assessment, including therapy modality. We established a prognostic score of survival in the chosen populations of NSCLC with MPE.

Our study included several limitations. First, this was a single‐center study spanning from 2010 to 2021, and the cohort may be representative in China. Second, antiangiogenic therapy is commonly used in Asia, and some geographical differences may exist.^18^ Additionally, this study lacks external validation, and future validation in a prospective cohort is needed. Finally, we should acknowledge the fact that NSCLC is a very heterogeneous disease nowadays with different mutations warranting different treatments. A one size fits all prognostic system for MPE has potential risks of over‐ or underestimating survival.

## 5 CONCLUSION

In conclusion, we developed a prognostic score to predict the survival of NSCLC patients with MPE. The CAIL score integrated clinical features and therapy modalities for predicting prognosis, which was shown to be simple to calculate and practical to use in clinical practice and useful for physicians in choosing the optimal management strategies.

## AUTHOR CONTRIBUTIONS

Tianyuan Li: collected data, analyzed data, and wrote the paper. Panwen Tian: designed study, performed study, collected data, analyzed data, and manuscript review. Qin Huang: literature search, collected data, analyzed data, and manuscript review. Hao Zeng: literature search, collected data, analyzed data, and manuscript review. Qi Wei: literature search, collected data, analyzed data, and manuscript review. Yalun Li: designed study, definition of intellectual content, literature search, collected data, analyzed data, manuscript preparation, manuscript editing, and manuscript review.

## CONFLICT OF INTEREST STATEMENT

None of the authors have a conflict of interest to disclose.

## ETHICS STATEMENT

The authors are accountable for all aspects of the work in ensuring that questions related to the accuracy or integrity of any part of the work are appropriately investigated and resolved. The present study was conducted in accordance with the principles of the Declaration of Helsinki (as revised in 2013). This study was approved by the Ethics Committee of West China Hospital (No. 2022‐1085). The need for patient informed consent was waived due to the retrospective nature of the study.

## Supporting information


**Figure S1.** The calibration curves of the nomogram for predicting overall survival (OS) in both the training and validation cohorts. The x‐axis represents the nomogram predicted probability, and the y‐axis represents the actual probability of OS. The red line indicates the performance of the nomogram, of which a closer fit to the gray line represents a better prediction. Calibration curves of the nomogram for predicting OS at (A) 6, (B) 12, and (C) 36 months in the training cohort. Calibration curves of the nomogram to predict OS at (D) 6, (E) 12, and (F) 36 months in the validation cohort.

**Table S1:** The CAIL score calculation.

## Data Availability

The data that support the findings of this study are available from the corresponding author upon reasonable request.
