# Supplementary figures and images for: Development and validation of the CAIL prognostic score in non‐small cell lung cancer patients with malignant pleural effusion
Source: Clin Respir J. 2023 Sep 18;17(11):1158–68. doi: 10.1111/crj.13700 (PMC10632079; doi:10.1111/crj.13700)

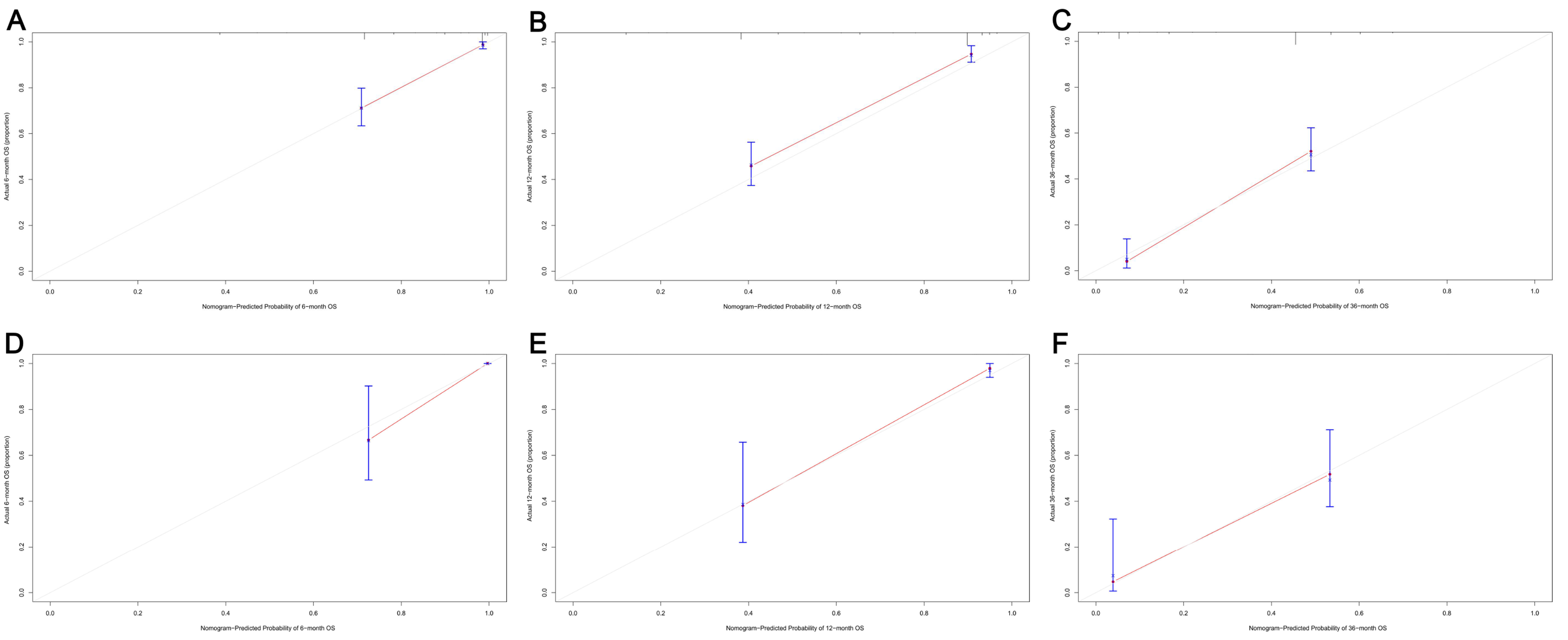

Supplement: Supplementary file 1 — Figure S1. The calibration curves of the nomogram for predicting overall survival (OS) in both the training and validation cohorts. The x‐axis represents the nomogram predicted probability, and the y‐axis represents the actual probability of OS. The red line indicates the performance of the nomogram, of which a closer fit to the gray line represents a better prediction. Calibration curves of the nomogram for predicting OS at (A) 6, (B) 12, and (C) 36 months in the training cohort. Calibration curves of the nomogram to predict OS at (D) 6, (E) 12, and (F) 36 months in the validation cohort. [file CRJ-17-1158-s002.tif]
