# Supplementary material for: Development and validation of the CAIL prognostic score in non‐small cell lung cancer patients with malignant pleural effusion
Source: Clin Respir J. 2023 Sep 18;17(11):1158–68. doi: 10.1111/crj.13700 (PMC10632079; doi:10.1111/crj.13700)
Supplement: Supplementary file 2 — Table S1: The CAIL score calculation. [file CRJ-17-1158-s001.docx]

| **Supplementary Table 1: The CAIL score calculation** | | |
| --- | --- | --- |
|  | Variable | Score |
| C | ECOG PS |  |
|  | 0 | 0 |
|  | 1 | 40 |
|  | 2 | 60 |
|  | 3-4 | 100 |
| A | Antiangiogenic therapy |  |
|  | No | 0 |
|  | Yes | 15 |
| I | Immunotherapy |  |
|  | No | 0 |
|  | Yes | 10 |
| L | Fluid LDH, IU/L |  |
|  | <1500 | 0 |
|  | ≥1500 | 35 |
| **Risk categories** | **Total score** |  |
| Low risk | 0-55 |  |
| Moderate risk | 56-105 |  |
| High risk | 106-160 |  |

ECOG PS = Eastern Cooperative Oncology Group performance score; LDH = lactate dehydrogenase
